# Activated hepatic stellate cells promote epithelial-to-mesenchymal transition in hepatocellular carcinoma through transglutaminase 2-induced pseudohypoxia

**DOI:** 10.1038/s42003-018-0177-5

**Published:** 2018-10-25

**Authors:** Hui Ma, Liqi Xie, Lan Zhang, Xin Yin, Hucong Jiang, Xiaoying Xie, Rongxin Chen, Haojie Lu, Zhenggang Ren

**Affiliations:** 1 Liver Cancer Institute, Zhongshan Hospital, Fudan University, 136 Yi Xueyuan Road, Shanghai, 200032 China; 2 Department of Chemistry and Institutes of Biomedical Sciences of Shanghai Medical School, Fudan University, 138 Yi Xueyuan Road, Shanghai, 200032 China

## Abstract

Activation of hepatic stellate cells reportedly contributes to progression of hepatocellular carcinoma (HCC). Herein, we use quantitative proteomics and ingenuity pathway analysis to show that transglutaminase 2 (TGM2) is upregulated in the course of activated hepatic stellate cells promoting epithelial-mesenchymal transition (EMT) in HCC-derived cells both in vivo and in vitro. Mechanistically, activated hepatic stellate cells promote TGM2 upregulation in HCC cells through inflammatory signalling; and TGM2-induced depletion of von Hippel-Lindau (VHL) protein, a key molecule in the degradation of hypoxia inducible factor-1a (HIF-1a) under normoxia, then causes HIF-1a to accumulate, thereby producing a pseudohypoxic state that promotes EMT in HCC cells. These findings suggest that the promotion of EMT in HCC cells by activated hepatic stellate cells is mediated by pseudohypoxia induced via TGM2/VHL/HIF-1a pathway.

## Abstract (web summary)

Hui Ma et al. report a new mechanism by which activated hepatic stellate cells promote the epithelial-to-mesenchymal transition (EMT) in hepatocellular carcinoma. They find that transglutaminase 2 is upregulated by activated hepatic stellate cells via inflammatory signalling, which leads to a pseudohypoxic state promoting EMT.

## Introduction

Hepatocellular carcinoma (HCC) is the fifth most common tumour worldwide and the second most common cause of cancer-related deaths^1^. As critical elements of the HCC microenvironment, activated hepatic stellate cells play central roles in chronic inflammation and subsequent reactive hepatic desmoplasia. Recently they have been found to stimulate growth, migration, and invasion of HCC cells, as several published studies indicate^2–4^. However, crosstalk between HCC cells and hepatic stellate cells pertaining to hepatic stellate cells activation and the promoting of HCC progression is still poorly understood.

The epithelial-mesenchymal transition (EMT), wherein epithelial cells depolarise, lose their cell–cell contacts, and acquire elongate, fibroblast-like morphology, is a potential mechanism by which tumour cells develop metastatic properties^5^. Functional implications of EMT include enhanced mobility, invasion, and resistance to apoptotic stimuli^5,6^. Although it has been noted that molecules secreted by hepatic stellate cells promote EMT in HCC cells, enabling migration and invasion, most studies have focused solely on singular hepatic stellate cell-secreted proteins and their roles in this regard; whereas few have investigated key molecules and pathways therein, using whole protein analysis of HCC cells once stimulated by hepatic stellate cells. The latter may reveal a global mechanism of malignant biologic behaviour in HCC, generating more desirable targets of anti-tumour therapy.

Mass spectrometry-based proteomics is a revolutionary technology allowing rapid identification and accurate quantification of thousands of proteins within a complex biological specimen^7^. Comparative proteomic analysis may thus provide an overview of dynamic changes promoted in HCC cells by hepatic stellate cells. Bioinformatics analysis of known and predicted protein–protein interactions can be used to cluster functional data and further characterise roles of differentially expressed proteins.

Transglutaminase 2 (TGM2) belongs to the family of transglutaminase enzymes and is a calcium-dependent cross-linking enzyme that catalyses protein modifications via transamidation, facilitating the formation of lysine combinations or polyaminated proteins in the presence of calcium^8^. TGM2 has been implicated in various biological functions, including differentiation of cells, extracellular matrix (ECM) stabilisation, and cell migration^8^. Recent studies have confirmed that TGM2 induces EMT and thus may contribute to acquired drug resistance in colon, breast, and gastric cancer cells; and increased expression of TGM2 appears to drive glycolytic metabolism in cells of breast and renal cancers^9–13^.

Findings of the present study confirm the phenomenon wherein activated hepatic stellate cells promote EMT in HCC cells both in vivo and in vitro. Through quantitative proteomics and ingenuity pathway analysis (IPA), we have shown that TGM2 is clearly upregulated as a result, leading to a pseudohypoxic state. This pseudohypoxia is due to enhanced hypoxia inducible factor-1a (HIF-1a) stability under normoxic conditions^14^ and TGM2-induced depletion of von Hippel-Lindau (VHL) protein, a key molecule in the degradation of HIF-1α^15^. This is the first evidence to our knowledge that promotion of EMT in HCC cells by activated hepatic stellate cells is mediated by pseudohypoxia induced via TGM2/HIF-1a pathway, demonstrating that TGM2 is a therapeutic target linked to inflammatory effects and the pseudohypoxic microenvironment of HCC.

## Results

### Activated hepatic stellate cells promote HCC cells EMT

**Fig. 1:**
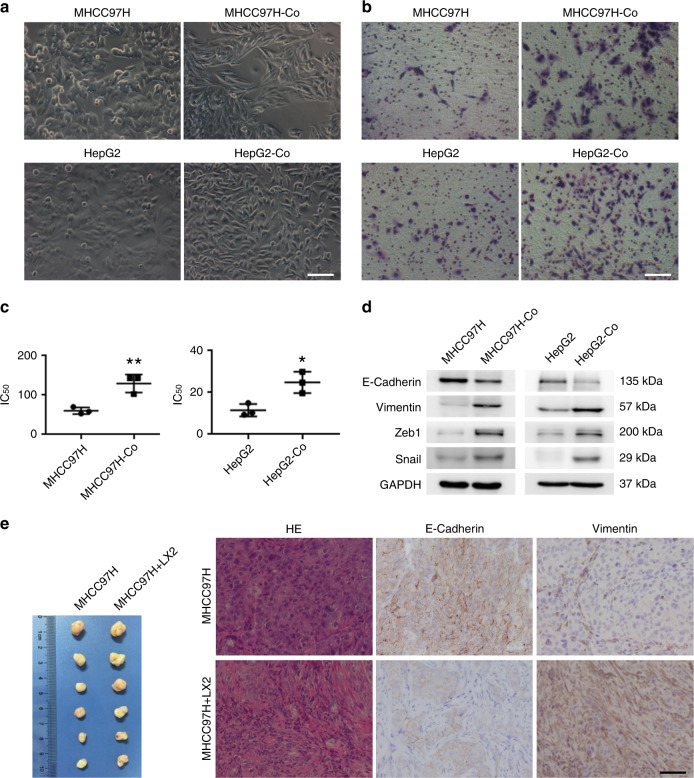
Activated hepatic stellate cells promote EMT in HCC cells in vivo and in vitro. **a** Morphologic changes in HCC cells (MHCC97H and HepG2) after co-culture with the hepatic stellate cells LX2 cells (MHCC97H-Co, HepG2-Co) observed under phase-contrast microscope (scale bar, 50 μm); **b** Invasiveness of HCC cells promoted after co-culture with hepatic stellate cells (scale bar, 50 μm); **c** Resistance to cisplatin in HCC cells promoted after co-culture with hepatic stellate cells (*P < 0.05, **P < 0.01, mean ± SEM); **d** Lower E-cadherin and higher Vimentin, Zeb1, and Snail expression levels in HCC cells co-cultured with hepatic stellate cells (western blotting); and **e** Lower E-cadherin and higher Vimentin expression levels in tumours spawned by combined injection of HCC cells and hepatic stellate cells into nude mice. Haematoxylin and eosin (HE)-stained images and immunostaining of E-cadherin and Vimentin are shown (scale bar, 50 μm). Experiments were repeated at least three times in triplicate

We optimised a co-culture system for in vitro use in this study, providing a physiologic milieu for interaction between HCC cells and activated hepatic stellate cells. Compared with control HCC cells, those co-cultured with an activated hepatic stellate cell line morphed into more spindle-like mesenchymal shapes, losing epithelial hallmarks of HCC cells (Fig. 1a). They also exhibited greater invasion and resistance to cisplatin (Fig. 1b, c), expressing less E-cadherin and more vimentin in support of a mesenchymal phenotype and expressing more EMT transcriptional factor Snail and Zeb1 (Fig. 1d and Supplementary Fig. 1)^5^. Upon subcutaneous injection of HCC cells alone or with hepatic stellate cells into nude mice, the HCC cells co-inoculated with hepatic stellate cells (vs. HCC cells alone) were associated with reduced E-cadherin expression and increased vimentin expression in vivo (Fig. 1e). Human c-Met expression helps to distinguish HCC cells and hepatic stellate cells, as the HCC cell line MHCC97H is known to highly express c-Met oncogene (Supplementary Fig. 2). These findings indicate that activated hepatic stellate cells promote EMT in HCC cells in vivo and in vitro.

### Activated hepatic stellate cells promote TGM2 upregulation in HCC cells

To identify proteins in HCC cells that are implicated in the promotion of EMT by hepatic stellate cells, an integrated approach, consisting of whole-cell protein digestion, stable isotope dimethyl labelling, LC-MS/MS analysis, and database screening was implemented to comparatively profile protein expression in MHCC97H cells, with or without activated hepatic stellate cell line LX2 cells co-culture. In three independent experiments, 106 and 70 proteins were up- or downregulated, respectively, in MHCC97H-Co cells, compared with control MHCC97H cells (Supplementary Tables 1, 2). The accuracy of mass spectra results was validated by western blotting of NDRG1, TGM2, and STAT2 (Supplementary Fig. 3).

**Fig. 2:**
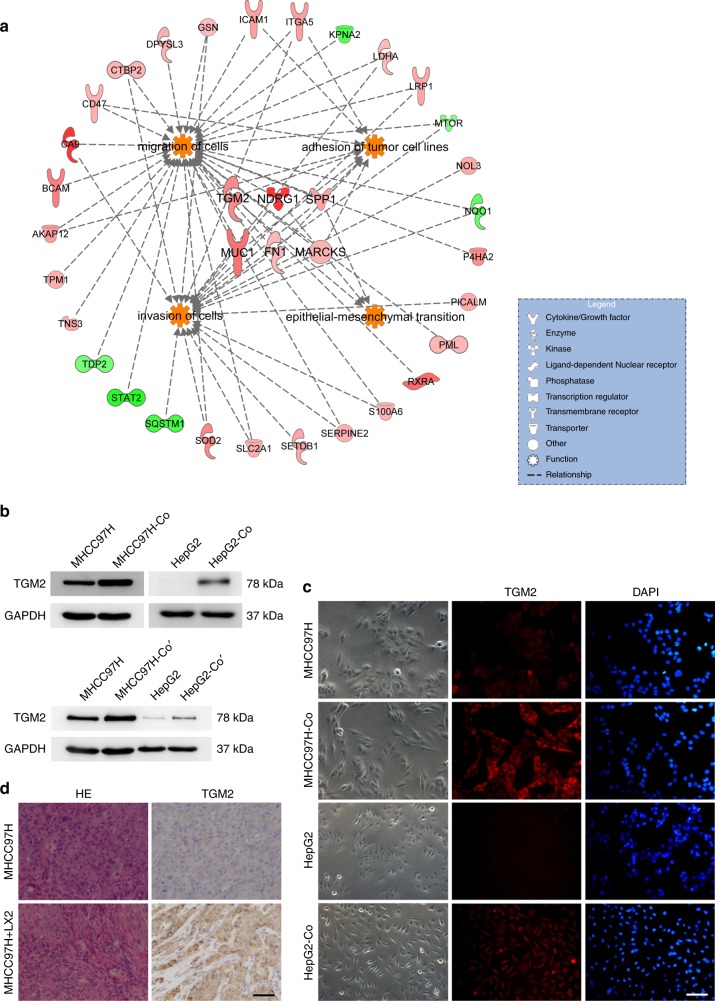
Activated hepatic stellate cells promote TGM2 upregulation in HCC cells in vivo and in vitro. **a** Thirty-five differentially expressed proteins identified in relation to EMT in MHCC97H-Co cells and resultant networks, analysed via Ingenuity Pathways Analysis software; **b** Heightened TGM2 expression in HCC cells after co-culture with LX2 cells (MHCC97H-Co, HepG2-Co) and activated human hepatic stellate cells (MHCC97H-Co’, HepG2-Co’), respectively (western blotting); **c** Heightened TGM2 expression (red) in MHCC97H-Co and HepG2-Co shown by immunofluorescence, with DAPI (blue) nuclear staining (scale bar, 50 μm); and **d** Heightened TGM2 expression in tumour cells following combined injection of MHCC97H and LX2 cells into nude mice. HE-stained images and immunostaining of TGM2 are shown (scale bar, 50 μm). Experiments were repeated at least three times in triplicate

A comprehensive view of the roles played by differentially expressed proteins in EMT was pursued, adopting IPA to group these proteins into functional networks and canonical pathways related to EMT and to determine altered proteins or pathways in MHCC97H cells after co-culture with LX2 cells, all reliant upon the underlying biologic evidence from the curated Ingenuity Pathways Knowledge Base. Functional clusters, including proteins involved in migration of cells, invasion of cells, adhesion of tumour cell lines, and EMT, were drawn from the database, and 35 differentially expressed proteins were identified relative to EMT in MHCC97H-Co cells (Fig. 2a). In accordance with previous studies and protein expression average ratios, the study was designed to investigate the biologic significance of TGM2 in activated hepatic stellate cells that promote EMT in HCC cells.

As confirmation of proteomic analysis, another five HCC cell line (MHCC97L, LM3, HepG2, Hep3B and Hu-7) and primary activated human hepatic stellate cells, respectively, were subjected to similar treatment. TGM2 was likewise upregulated in HCC cells after co-culture with activated hepatic stellate cells at both mRNA and protein levels (Fig. 2b, Supplementary Figs. 1, 4). The upregulated TGM2 largely assumed an intracytoplasmic location under immunofluorescence (Fig. 2c). We also investigated TGM2 expression in subcutaneous tumours after injecting HCC cells alone or in conjunction with hepatic stellate cells into nude mice. Accordingly, HCC cells co-inoculated with hepatic stellate cells (vs. HCC cells injected alone) demonstrated increased TGM2 expression in vivo (Fig. 2d).

Altogether, these findings constitute the first evidence to our knowledge that activated hepatic stellate cells promote TGM2 upregulation in HCC cells in vivo and in vitro, implicating TGM2 upregulation as the main driver of EMT in HCC cells.

### TGM2 upregulation promotes EMT in HCC cells

**Fig. 3:**
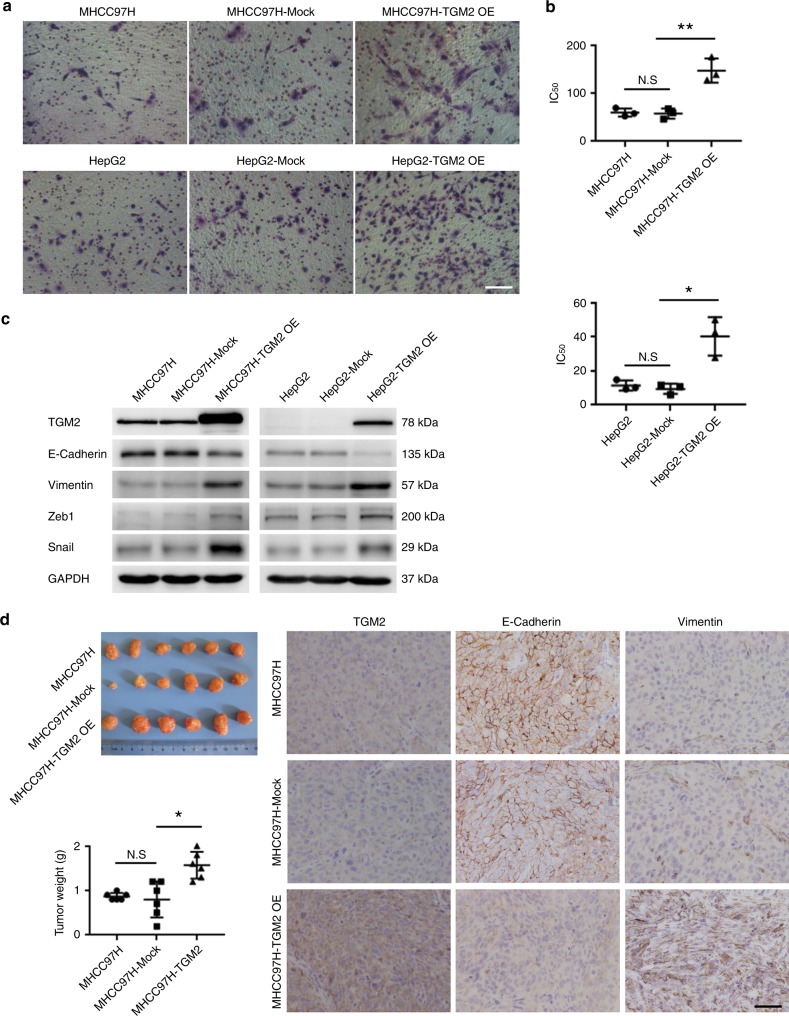
TGM2 upregulation promotes EMT in HCC cells in vivo and in vitro. **a** Enhanced invasiveness of HCC cells (MHCC97H and HepG2) due to lentivirus-mediated TGM2 overexpression (MHCC97H-TGM2 OE and HepG2-TGM2 OE), compared with two HCC cell lines and vector control (MHCC97H-Mock and HepG2-Mock) (scale bar, 50 μm); **b** Increased resistance to cisplatin in MHCC97H-TGM2 OE and HepG2-TGM2 OE cells, compared with MHCC97H-Mock and HepG2-Mock cells, respectively (*P < 0.05, mean ± SEM); **c** Lower E-cadherin and higher Vimentin, Zeb1, and Snail expression levels in MHCC97H-TGM2 OE and HepG2-TGM2 OE cells, compared with MHCC97H-Mock and HepG2-Mock cells, respectively (western blotting); **d** Subcutaneous tumours produced by injecting MHCC97H-TGM2 OE cells were heavier, compared with counterpart MHCC97H-Mock cell injections (*P < 0.05, mean ± SEM). Note lower E-cadherin and higher Vimentin expression levels in tumour cells produced by injecting MHCC97H-TGM2 OE cells into nude mice, compared with counterpart MHCC97H-Mock cell injections. Images of immunostained TGM2, E-cadherin, and Vimentin are shown (scale bar, 50 μm). Experiments were repeated at least three times in triplicate

The functional involvement of TGM2 upregulation in promoting EMT in HCC cells was subsequently studied by examining lentivirus-mediated TGM2 overexpression (OE) and vector controls in HCC cells (HCC-TGM2 OE and HCC-Mock). Compared with control HCC-Mock cells, HCC-TGM2 OE cells exhibited greater invasion and resistance to cisplatin in association with reduced E-cadherin expression and increased vimentin, Zeb1 and Snail expression (Fig. 3a–c and Supplementary Fig. 5). We subcutaneously injected HCC-TGM2 OE cells, HCC-Mock cells, and parent HCC cells into nude mice. The tumours arising from injected HCC-TGM2 OE cells (vs. inoculated HCC-Mock cells) had a greater mass, accompanied by reduced E-cadherin expression and increased vimentin expression (Fig. 3d). These results again implicate TGM2 upregulation as a driver of EMT in HCC cells in vivo and in vitro.

### TGM2 knockdown inhibits EMT in HCC cells

**Fig. 4:**
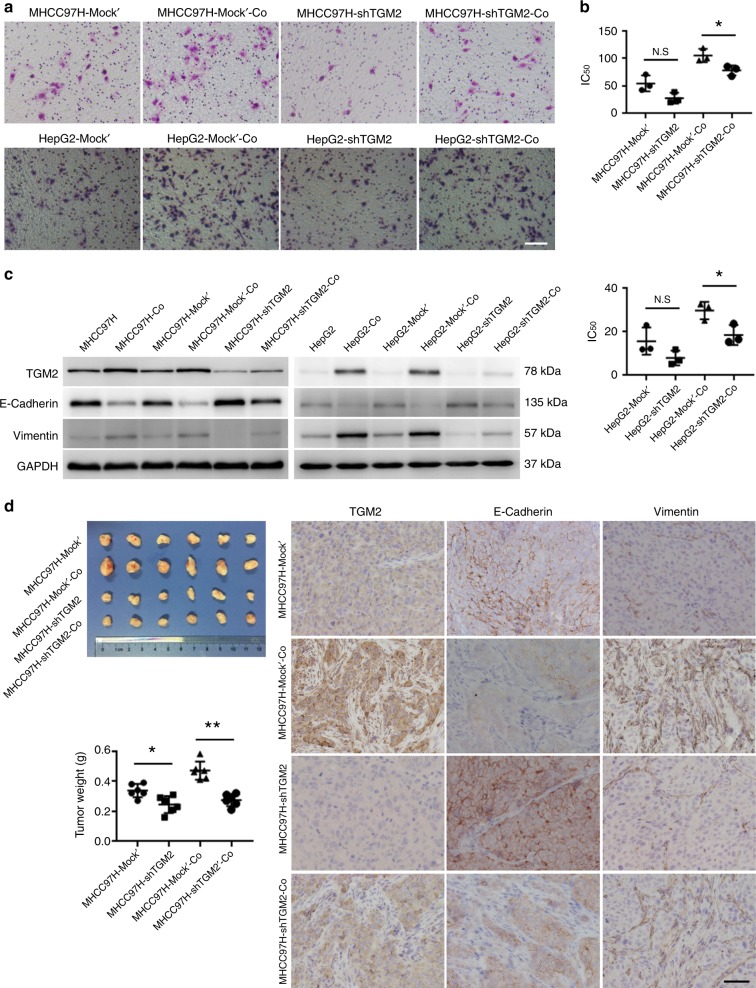
TGM2 knockdown inhibits HCC cells EMT promoted by activated hepatic stellate cells in vivo and in vitro. **a** Diminished invasiveness of HCC cells (MHCC97H and HepG2) after lentivirus-mediated TGM2 knockdown co-culturing with LX2 cells (MHCC97H-shTGM2-Co and HepG2-shTGM2-Co), compared with two HCC cell lines and vector control co-cultured with LX2 cells (MHCC97H-Mock’-Co and HepG2-Mock’-Co), respectively (scale bar, 50 μm); **b** Less resistance to cisplatin in MHCC97H-shTGM2-Co and HepG2-shTGM2-Co cells, compared with MHCC97H-Mock’-Co and HepG2-Mock’-Co cells, respectively (*P < 0.05, mean ± SEM); **c** Higher E-cadherin and lower Vimentin expression levels in MHCC97H-shTGM2-Co and HepG2-shTGM2-Co cells, compared with MHCC97H-Mock’-Co and HepG2-Mock’-Co cells, respectively (western blotting); and **d** Subcutaneous tumours following injection of MHCC97H-shTGM2 cells and LX2 cells were lighter compared with counterpart injections of MHCC97H-Mock’cells and LX2 cells (*P < 0.05, **P < 0.01, mean ± SEM). Note higher E-cadherin and lower Vimentin expression levels in tumour cells produced by injecting MHCC97H-shTGM2 cells and LX2 cells into nude mice, compared with counterpart injection of MHCC97H-Mock’ and LX2 cells. Images of immunostained TGM2, E-cadherin, and Vimentin are shown (scale bar, 50 μm). Experiments were repeated at least three times in triplicate

In questioning whether TGM2 upregulation is a necessary condition for promotion of EMT in HCC cells by activated hepatic stellate cells, we studied lentivirus-mediated TGM2 knockdown and vector controls in HCC cells (HCC-shTGM2 and HCC-Mock’). HCC-shTGM2 and HCC-Mock’ cells were co-cultured with LX2 cells (HCC-shTGM2-Co and HCC-Mock’-Co). Compared with control HCC-Mock’-Co cells, HCC-shTGM2-Co cells exhibited less invasion and resistance to cisplatin in association with increased E-cadherin expression and reduced vimentin expression (Fig. 4a–c). As controls, HCC-shTGM2 and HCC-Mock’cells underwent the same analysis. Compared with HCC-Mock’ cells, HCC-shTGM2 cells displayed less invasion and resistance to cisplatin (although not statistically significant), showing increased E-cadherin expression and reduced vimentin expression, (Fig. 4a–c). We injected HCC-shTGM2 and HCC-Mock’cells alone or with hepatic stellate cells into the subcutis of nude mice. Compared with control injections of HCC-Mock’ cells and hepatic stellate cells, the tumours produced by injecting HCC-shTGM2 cells and hepatic stellate cells were marked by significantly lighter weight, showing increased E-cadherin expression and reduced vimentin expression (Fig. 4d). Likewise, the tumours produced by injecting HCC-shTGM2 cells alone were significantly lighter and showed increased E-cadherin expression and reduced vimentin expression compared with injections of HCC-Mock’ cells alone (Fig. 4d). These results together imply that TGM2 knockdown inhibits promotion of EMT in HCC cells by activated hepatic stellate cells in vivo and in vitro.

### TGM2 upregulation leads to HIF-1a accumulation under normoxic conditions

**Fig. 5:**
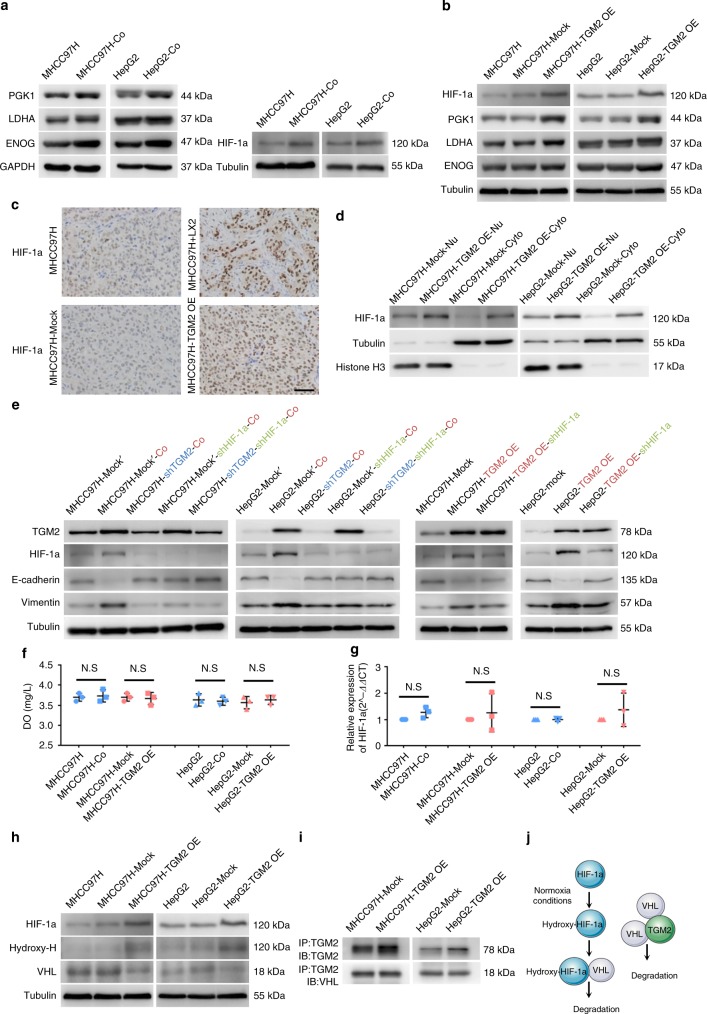
TGM2 upregulation results in the accumulation of HIF-1a under normoxic conditions promoting EMT in HCC cells: PGK1, LDHA, ENOG, and HIF-1a expression levels increased in HCC cells (MHCC97H and HepG2) co-cultured with LX2 cells (**a**) and increased in MHCC97H-TGM2 OE and HepG2-TGM2 OE cells compared with counterpart cells (western blotting). **b**, **c** Heightened HIF-1a expression in tumour cells following combined injection of MHCC97H and LX2 cells into nude mice, and following injection of MHCC97H-TGM2 OE cells, compared with counterpart cells injection (Immunohistochemistry, scale bar, 50 μm); **d** Heightened HIF-1a expression in both cytoplasm and nucleus of MHCC97H-TGM2 OE and HepG2-TGM2 OE cells, compared with MHCC97H-Mock and HepG2-Mock cells, respectively (western blotting); **e** Higher E-cadherin and lower Vimentin expression levels in HCC cells after plasmid-mediated HIF-1a knockdown co-culturing with LX2 cells (MHCC97H-Mock’-shHIF-1a-Co and HepG2-Mock’-shHIF-1a-Co), compared with two HCC cell lines subjected to plasmid control co-culturing with LX2 cells (MHCC97H-Mock’-Co and HepG2-Mock’-Co), respectively (western blotting). Note higher E-cadherin and lower Vimentin expression levels in MHCC97H-TGM2 OE and HepG2-TGM2 OE cells after plasmid-mediated HIF-1a knockdown (MHCC97H-TGM2 OE-shHIF-1a and HepG2-TGM2 OE- shHIF-1a), relative to two HCC plasmid control cell lines (MHCC97H-TGM2 OE and HepG2-TGM2 OE), respectively (western blotting); **f** Dot plots showing no significant difference in oxygen concentration of culture medium in HCC cells co-cultured with LX2 cells (MHCC97H-Co and HepG2-Co) and HCC cells alone or in HCC cells showing TGM2 upregulation (MHCC97H-TGM2 OE and HepG2-TGM2 OE) and control HCC cells (MHCC97H-Mock and HepG2-Mock), as registered by dissolved oxygen metres; **g** No significant difference in HIF-1a gene expression in HCC cells co-cultured with LX2 cells and in HCC cells alone, or in HCC cells showing TGM2 upregulation and control HCC cells (quantitative real-time polymerase chain reaction); **h** Higher hydroxy-HIF-1a and lower VHL expression levels in MHCC97H-TGM2 OE and HepG2-TGM2 OE cells, compared with MHCC97H-Mock and HepG2-Mock cells, respectively (western blotting); **i** Immunoprecipitation of MHCC97H and HepG2 cell lysates, with or without TGM2 upregulation, using anti-TGM2 antibody (confirmatory western blot using anti-TGM2 and anti-VHL antibodies); and **j** TGM2 binding and polymerisation of VHL result in degradative VHL depletion, causing HIF-1a to accumulate. Experiments were repeated three times in triplicate

Among the differentially expressed proteins identified through quantitative proteomics, several proteins critical in glycolysis (i.e., PGK1, LDHA, and ENOG) have proved to be upregulated in HCC cells after co-culture with activated hepatic stellate cells, despite an apparently normoxic physiologic milieu^16,17^. We have confirmed the upregulation of these proteins in HCC-Co cells by western blot (Fig. 5a). To our knowledge, PGK1, LDHA, and ENOG are all downstream proteins of HIF-1a, and under hypoxic conditions, HIF-1a upregulation promotes their expression^16,17^. We found levels of HIF-1a proteins in HCC-Co cells to be significantly higher than those of control HCC cells, respectively (Fig. 5a). To determine whether TGM2 upregulation regulates HIF-1a expression, HIF-1a, PGK1, LDHA, and ENOG expression levels were measured in HCC-TGM2 OE cells, as well as in corresponding control cells. Subsequently, expression levels of HIF-1a and two downstream proteins, LDHA and PGK1, were all increased in HCC-TGM2 OE cells, compared with control HCC-Mock cells (Fig. 5b). However, ENOG expression was not significantly increased in HCC cells showing TGM2 upregulation (Fig. 5b). HIF-1a expression was also detected in the subcutaneous tumour implants, including those generated by HCC cells alone, HCC cells with hepatic stellate cells, HCC-Mock cells alone, and HCC-TGM2 OE cells alone. Consequently, in tumour implants devoid of necrosis due to ischaemia and hypoxia, HIF-1a expression was higher in tumour cells following combined injection of HCC cells and hepatic stellate cells, compared with injection of HCC cells alone, and higher HIF-1a expression was evident in tumour cells after implanting HCC-TGM2 OE cells alone, compared with implantation of HCC-Mock cells alone. In addition, we observed higher levels of HIF-1a expression at both cytoplasmic and nuclear location in subcutaneously implanted tumour cells of HCC cells and hepatic stellate cells together or HCC-TGM2 OE cells alone, compared with respective control groups (Fig. 5c). To confirm this phenomenon, HIF-1a expression was assayed in both cytoplasmic and nuclear locations of HCC-TGM2 OE and HCC-Mock cells by western blot. Indeed, expression of HIF-1a was quantitatively elevated in both cytoplasm and nucleus of HCC-TGM2 OE cells, compared with HCC-Mock cells.

To address the mechanism involved in promotion of EMT by HIF-1a under hepatic stellate cells co-culturing conditions, plasmid-mediated HIF-1a knockdown and plasmid controls were undertaken in HCC-Mock’, HCC-shTGM2, and HCC-TGM2 OE cells. The outcomes indicated that EMT was clearly weakened in HCC cells with HIF-1a knockdown co-culturing with hepatic stellate cells, the extent of which was similar to of TGM2 knockdown alone or combined, and EMT was also weakened in HCC-TGM2 OE cells with HIF-1a knockdown (Fig. 5e).

To study the mechanism by which TGM2 induces increases in HIF-1a protein levels, both gene expression and protein stability of HIF-1a were explored. First, there were no significant differences in oxygen concentrations of the media used for HCC cells and hepatic stellate cells co-culturing, compared with HCC cells cultured alone, or in culturing of HCC cells with upregulated TGM2, compared with control HCC cells (Fig. 5f). In addition, there were no significant differences in HIF-1a gene expression by HCC cells co-cultured with hepatic stellate cells, compared with HCC cells cultured alone or by HCC cells with upregulated TGM2, compared with control HCC cells (Fig. 5g). Therefore, TGM2 was believed to affect the stability of HIF-1a protein, key proteins in the HIF-1a degradation pathway, specifically VHL, were assayed in HCC cells showing TGM2 upregulation and control HCC cells by western blot. In HCC cells showing TGM2 upregulation, accumulated hydroxy-HIF-1a increased, whereas accumulated VHL declined, compared with control HCC cells (Fig. 5h and Supplementary Fig. 6). Pertinent research has shown that TGM2 can bind and polymerise VHL, directly depleting VHL through ubiquitination and proteasomal degradation^15^, so immunoprecipitation was performed to confirm the binding of TGM2 and VHL in HCC cells showing TGM2 upregulation (Fig. 5i).

In summary, TGM2 upregulation results in the accumulation of HIF-1a under normoxic conditions, and it is through this mechanism of hepatic stellate cells activation that EMT is promoted in HCC cells.

### TGM2 is upregulated in HCC cells via inflammatory signalling

**Fig. 6:**
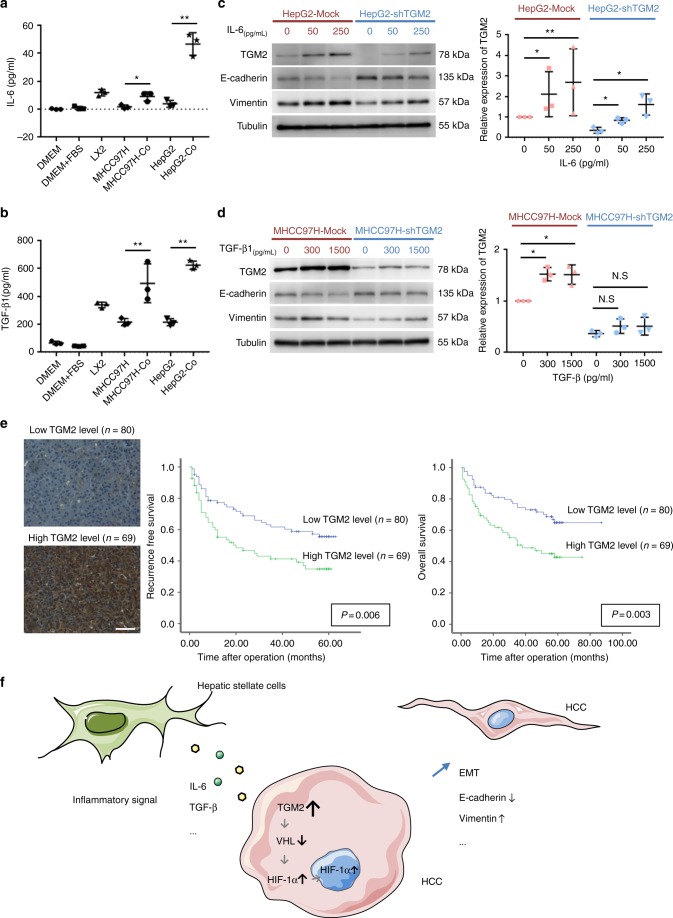
Activated hepatic stellate cells promote TGM2 upregulation in HCC cells through inflammatory signals and heightened TGM2 expression in HCC tissue bodies poorly for clinical outcomes: **a**, **b** Levels of IL-6 and TGF-β1 released in supernatant of co-cultured HCC cells and hepatic stellate cells proved significantly higher than levels produced by HCC cells alone (enzyme-linked immunosorbent assay) (*P < 0.05, **P < 0.01, mean ± SEM, experiments were repeated three times in triplicate); **c**, **d** TGM2 upregulation in HCC cells after IL-6 treatment (0, 50, and 250 pg/mL) and TGF-β1 treatment (0, 300, and 1500 pg/mL), with TGM2 knockdown inhibiting rise in E-cadherin and fall in Vimentin expression levels promoted in HCC cells by IL-6 and TGF-β1 treatment (*P < 0.05, **P < 0.01, mean ± SEM, experiments were repeated three times in triplicate); **e** Heightened (vs. low-level) postoperative TGM2 expression linked with significantly worse recurrence-free and overall survival in patients with HCC (scale bar, 50 μm). Log-rank *P*-values are shown; and **f** Schematic illustration of EMT promotion in HCC cells by activated hepatic stellate cells through TGM2 upregulation

The cause of TGM2 upregulation in HCC cells co-cultured with activated hepatic stellate cells was examined. TGM2 upregulation in cells is reportedly linked to inflammatory signals^18^. IL-6 and TGF-β1 are important inflammatory mediators that have been implicated in tumour metastasis and the triggering of EMT, and they have been identified in proteins secreted by activated hepatic stellate cells^10,19,20^. According to our observations, levels of IL-6 and TGF-β1 released into the supernatant of our HCC cells and hepatic stellate cells co-culture system were significantly higher than levels associated with HCC cells alone (Fig. 6a, b). We also assayed TGM2 in HCC cells after IL-6 and TGF-β1 treatment, discovering that it was upregulated, compared to levels found in control HCC cells (Fig. 6c, d). Furthermore, TGM2 knockdown inhibited EMT in HCC cells promoted by IL-6 and TGF-β1 treatment (Fig. 6c, d).

### High TGM2 expression in HCC tissue is associated with poor clinical outcomes

Using tissue microarrays, immunohistochemistry was performed to gaugeTGM2 protein levels in tumour specimens from 149 patients with HCC. In scored samples, 69 were considered high, and the remaining 80 were considered low. After a median follow-up of 47 months (range, 1–64 months), patients with high (vs. low) TGM2 levels displayed significantly worse recurrence-free (*P* = 0.006) and overall (*P* = 0.003) survival rates (Fig. 6e).

In addition, gene expression analysis using The Cancer Genome Atlas (TCGA) database showed that there was no significance between TGM2 mRNA levels in HCC tissues and the adjacent normal tissues, but the TGM2 expression in HCC to adjacent normal tissues ratio was significantly higher in TNM stage II/III in comparison to in stage I patients (Supplementary Fig. 7). There was a positive correlation between TGM2 and vimentin expression (Supplementary Fig. 7, *r*^2^ = 0.276, *p* < 0.01), but no significant correlation between TGM2 and E-cadherin expression in 371 HCC tissues from TCGA (*r*^2^ = 0.047, *p* = 0.366).

## Discussion

Although it has been reported that molecules secreted by hepatic stellate cells promote EMT in HCC cells, unleashing their migratory and invasive potential, the complex crosstalk between HCC cells and hepatic stellate cells is still poorly understood^2–4^. Most studies have targeted singular proteins secreted by hepatic stellate cells, characterising their roles in the progression of HCC. However, few have investigated the full gamut of protein changes in HCC cells (once stimulated by hepatic stellate cells) to perhaps unearth a global mechanism for their malignant biologic behaviour and better suited targets of anti-tumour therapy.

In the present study, the prominent role of TGM2 in activated hepatic stellate cells promoting EMT of HCC cells, has been demonstrated through quantitative proteomics and IPA and confirmed by in vitro and in vivo experimentation. Additionally, activated hepatic stellate cells seem to promote TGM2 upregulation in HCC cells through inflammatory signals, with TGM2 upregulation causing HIF-1a accumulation under normoxic conditions, and HIF-1a-mediated pseudohypoxia promoting EMT in HCC cells (Fig. 6f). This newly described chain of events represents a distinctive path through the HCC microenvironment in which inflammatory signals are converted to hypoxic signals by TGM2 upregulation, even under normoxic conditions. Inhibition of TGM2 upregulation thus promises a unique paradigm in HCC treatment, aimed at suppressing tumour progression by a remodelling of tumour milieu.

EMT is an important initiating event in cancer metastasis, stemness, and drug resistance^5,6^. Studies have shown that TGM2 influences EMT both in cancerous and fibrotic states^9–11,21^. Herein, we have shown that TGM2 upregulation promotes EMT in HCC cells, and that TGM2 knockdown inhibits the promotion of EMT by activated hepatic stellate cells in HCC cells, confirming the importance of TGM2 in tumour progression. These findings are aligned with previous studies of colon, breast, and gastric cancer cells, all indicating that TGM2 is pivotal in inducing EMT^9–11^. Unfortunately, other conclusions reached in terms of the mechanism involved have varied, implicating Wnt/β-catenin, ERK1/2, AKT, and NF-κB pathways. Still, the overriding process is likely to emerge as the endpoint of a high-throughput and integrated strategy. Through our quantitative proteomics approach, we have discovered that a pseudohypoxic state induced in the microcosm of HCC appears to activate hepatic stellate cells and promote EMT, which may then regulate or affect any of the pathways above^22,23^. It is also curious that HCC cells upregulate HIF-1a by initiating hypoxic responses under conditions of normoxia. Mechanistically, TGM2 consumes VHL, a critical molecule in the degradation of HIF-1a under normoxia^15^, and the resultant falsely hypoxic state is attributable to accumulation of HIF-1a.

The observation that hepatic stellate cells induce upregulation of TGM2, producing high basal expression of HIF-1α, is of immense importance, suggesting a major role for TGM2 in inflammation-regulated cancer progression. Expression of HIF-1α is considered a negative prognostic factor, given its implications in processes including chemoresistance, angiogenesis, invasiveness, metastasis, resistance to cell death, altered metabolism, and genome stability^22,23^. Because it is unstable under normoxic conditions, the effects HIF-1 are typically consigned to hypoxic disorders only. Our data have demonstrated that in HCC cells, HIF-1α stability is governed by hepatic stellate cells through TGM2 upregulation. There are vast scientific and clinical ramifications attached to such interaction between HCC cells and hepatic stellate cells.

Earlier reports have indicated that HCC cells can also stimulate the activation of hepatic stellate cells^24^. As critical elements of the HCC microenvironment, activated hepatic stellate cells play central roles in chronic inflammation and subsequent reactive hepatic desmoplasia; are known to secrete growth factors and cytokines; and in turn, stimulate the growth, migration, and invasion of HCC cells, according to several recently published studies^2–4^. Cytokines related to inflammation, such as IL-6 and TGF-β1, have been identified as secreted proteins of activated hepatic stellate cells^20^, also having been implicated in tumour metastasis and the triggering of EMT^10,19^. Additionally, inflammatory signals have been shown to upregulate TGM2 expression via NF-κB activation in tumour cells^10,18^. According to our results, activated hepatic stellate cells promote TGM2 upregulation, with ensuing EMT in HCC cells responsible for secretion of cytokines related to inflammation, thus offering a somewhat full depiction of cell–cell interaction in this setting.

TGM2 has been implicated in various biologic functions, including differentiation of cells, ECM stabilization, and cell migration^8^. The clinical significance of TGM2 has been followed with keen interest in recent years. Our efforts have proven that high TGM2 expression in HCC tissue is associated with lower rates of recurrence-free and overall survival. Others have similarly shown that TGM2 expression is a prognostic marker in colon cancer and non-small cell lung cancer; and in proteomic profiles, TGM2 upregulation in HCC correlates with early recurrence^25–27^.

In summary, the present data show that activated hepatic stellate cells promote EMT in HCC cells, mediated by a pseudohypoxic state that is induced via TGM2/HIF-1a pathway. Furthermore, TGM2 appears to be a central signal linking inflammatory effects and pseudohypoxia in the HCC microenvironment.

## Methods

### Cell sources and culture techniques

The human HCC cell line MHCC97L, MHCC97H, LM3 was established by the Liver Cancer Institute of Fudan University, with the same genetic background and gradually increasing metastatic potential. The human HCC cell line HepG2, Hep3B, Hu-7, and the human stellate cell line LX2 were obtained from the State Key Laboratory of Oncogenes and Related Genes, Cancer Institute, Shanghai Jiaotong University. Hep3B cells were cultured in Modified Eagle’s medium (MEM; Gibco) containing 10% v/v foetal bovine serum (FBS, Gibco), other cell lines were cultured in Dulbecco’s modified Eagle’s medium (DMEM; Gibco) containing 10% v/v foetal bovine serum (FBS, Gibco). Primary human hepatic stellate cells (gifted by RX Chen) were maintained in complete stellate cell medium^28^. Long-term culture of primary hepatic stellate cells in polystyrene dishes can recapitulate the features of activated hepatic stellate cells and has been widely accepted as an in vitro model for hepatic stellate cells activation studies^7^. All the cell lines passed the conventional tests of cell line quality control methods (e.g., morphology, isoenzymes, mycoplasma). All cell cultures were undertaken in a temperature-controlled (37 °C) humidified atmosphere (5% CO_2_). CTNNB1, P53, and VHL expression were assessed in the 6 HCC cell lines by western blot (Supplementary Fig. 8).

### Lentivirus, plasmids, and transfection

Lentivirus/GV287-TGM2 OE (Ubi-TGM2-3FLAG-SV40-EGFP), Lentivirus/GV115-shTGM2 (hU6-shTGM2-CMV- EGFP), and corresponding control lentiviruses (GV287 and GV115, respectively) were purchased (GeneChem, Shanghai, China). TGM2 OE was constructed from full-length TGM2 cDNA, and shTGM2 was generated from shRNA (5′-GCAGTGACTTTGACGTCTT-3′) targeting the TGM2 cDNA sequence. Plasmid/GV248-shHIF-1a (hU6-shHIF-1a-Ubi-EGFP-IRES-puromycin) and control plasmid/GV248 were also purchased (GeneChem). shRNA (5′-GATGAAAGAATTACCGAAT -3′) was used to target the HIF-1a cDNA sequence, using a non-target shRNA as control. All transfections were conducted in accord with manufacturer-supplied protocols. Oligo sequences are also shown in Table 1.

**Table 1:** Oligo sequences used in this study

shRNA		
shTGM2	5′- GCAGTGACTTTGACGTCTT -3′	Targets TGM2 cDNA sequence
Plasmid/GV248-shHIF-1a	5′- GATGAAAGAATTACCGAAT -3′	Targets HIF-1a cDNA sequence
**qRT-PCR**	**Forward**	**Reverse**
TGM2	5′- ACCGCTGAGGAGTACGTCTG -3′	5′- AAAGGCTCCAGGTTGAGGTT -3′
HIF-1a	5′- TGCAACATGGAAGGTATTGC -3′	5′- GCACCAAGCAGGTCATAGGT -3′
18S RNA	5′- CAGCCACCCGAGATTGAGCA -3′	5′- TAGTAGCGACGGGCGGTGTG -3′

### Human HCC specimens

Between January 2008 and December 2009, specimens were obtained from 149 patients with histologically confirmed HCC who underwent surgical resection at Zhongshan Hospital of Fudan University. The tissues were fixed in neutral formalin within 30 min of resection. The study was approved by the Zhongshan Hospital Research Ethics Committee (NO. 2007-17). Informed consent was obtained according to the committee’s regulations.

### Co-culture system and microscopy

The co-culture system incorporated a six-well plate containing HCC cells in DMEM with 10% FBS for 12 h before the experiment^24^. Hepatic stellate cells were cultured in 3-μm cell culture inserts (Millipore Sigma, Billerica, MA, USA) incubated for 72 h in six-well dishes with MHCC97H cells (HepG2 cells, 48 h). Once completed, the morphology of HCC cells was examined by light microscopy (Olympus, Tokyo, Japan). HCC cells and the conditioned media were utilised in subsequent experiments.

### Protein extraction, isotopic dimethylation labelling and liquid chromatography tandem mass spectrometry (LC-MS/MS) analysis

Whole-cell lysates of MHCC97H cells, with or without co-cultured LX2 cells, were extracted. Isotopic dimethylation labelling and LC-MS/MS experiments were then performed. Details are provided in the Supplementary Material.

### Bioinformatics analysis of differentially expressed proteins

Bioinformatics analysis of differentially expressed proteins was enabled by IPA software (June 2016 release; Qiagen Silicon Valley, Redwood City, CA, USA). Details are provided in the Supplementary Material.

### Transwell migration assay

Cell invasion was assessed using transwell migration assays in conjunction with co-cultured HCC cells^29^. Matrigel (50 μL; BD Biosciences, Franklin Lakes, NJ, USA) diluted in DMEM (1:8) was added to each well 4 h before cells were seeded onto membranes (Boyden chambers; Millipore Sigma). Cells in serum-free DMEM (5 × 10^4^) were then seeded onto membranes (8.0-μm pore size) in each upper well chamber of a 24-well plate. DMEM containing 10% FBS was added to each lower chamber. After 24 h, cells reaching membrane undersides were stained (Giemsa) and counted at 200x magnification.

### CCK-8 assay

Briefly, HCC cell lines were seeded onto a 96-well plate (3 × 10^3^ cells/well) 12 h prior to treatment with various concentrations (0–512 μg/ mL) of cisplatin (Sigma-Aldrich, St. Louis, MO, USA)^29^. The cells were grown in a final 100-mL volume of culture medium per well. CCK-8 labelling reagent (10 μL; Dojindo Molecular Technologies Inc, Kumamoto, Japan) was added to each well and incubated for 1 h. Absorbance of the formazan product was measured at 450 nm. To achieve significance in quantitative analysis, experiments were repeated at least three times.

### Animal experimentation

All animal experimentation protocols were approved by the Ethical Committee on Animal Experiments of the Animal Care Committee at Fudan University, and all testing complied with guidelines of the Shanghai Medical Experimental Animal Care Commission. Animals were maintained under specific pathogen-free conditions, making all efforts to minimise any suffering. All were purchased (SLAC Laboratory Animal Co Ltd, Shanghai, China), obtaining male BALB/c nu/nu mice (weights, 18–20 g) at 4 weeks of age. Each of 12 mice was injected subcutaneously (upper right flank) with a suspension (5 × 10^6^) of MHCC97H cells alone (*n* = 6) or with LX2 cells (5 × 10^6^, 1:1, *n* = 6). Another 18 mice were similarly injected with suspensions (5 × 10^6^) of MHCC97H cells (*n* = 6), MHCC97H-Mock cells (*n* = 6), or MHCC97H-TGM2 OE cells (*n* = 6); and the process was repeated in 24 more mice, injecting MHCC97H-Mock’ cells alone (5 × 10^6^, *n* = 6) or with LX2 cells (5 × 10^6^, 1:1, *n* = 6) and then MHCC97H-shTGM2 cells alone (5 × 10^6^, *n* = 6) or with LX2 cells (5 × 10^6^, 1:1, *n* = 6). The experiments were continued for 20 days. Tumour growth kinetics were recorded every 2 days after injection. Animals were sacrificed once the tumours were removed, weighing and fixing the tissue in 10% formalin or freezing specimens in liquid nitrogen.

### Preparation of whole-cell lysates, subcellular fractionation, and western blot

Briefly, whole-cell lysates were extracted using RIPA lysis buffer (Beyotime Biotechnology, Jiangsu, China) plus phenylmethylsulfonyl fluoride (PMSF) and a phosphatase inhibitor (Roche, Basel, Switzerland) at 4 °C^30^. Cytosol and nuclear extracts were prepared using NE-PER Nuclear and Cytoplasmic Extraction Reagents (Pierce Biotechnology, Rockford, IL, USA). Protein samples were separated via 10% sodium dodecyl sulfate-polyacrylamide gel electrophoresis (SDS-PAGE) gel and transferred onto polyvinylidene difluoride membranes (Millipore Sigma). The membranes were blocked in 5% bovine serum albumin (Beyotime Biotechnology) for 2 h, then washed and incubated with primary antibodies, at concentrations specified by the manufacturer, overnight at 4 °C. After washing, the membrane was incubated with appropriate HRP-conjugated secondary antibodies (Santa Cruz Biotechnology, Dallas, TX, USA) and examined using enhanced chemiluminescence (Pierce Biotechnology). The proteins were quantified by densitometry, using ImageJ software (National Institutes of Health, Bethesda, MD, USA), and normalised to relative internal standards. All experiments were performed in triplicate. A listing of primary antibodies used appears in the Supplementary Table 3. Original blot images corresponding to immunoblots in the main figures are presented in Supplementary Fig. 9.

### Immunoprecipitation

Briefly, whole-cell lysates were extracted using NP-40 lysis buffer (Beyotime Biotechnology) with phosphatase inhibitor (Roche) at 4 °C^31^. Total protein (1 mg) was incubated with TGM2 antibody (2 μg) for 18 h. The complex was then collected using Agarose A/G protein beads (Millipore Sigma) for 4 h and washed in cold PBS buffer containing protease and phosphatase inhibitors.

### Immunofluorescence

Expression of TGM2 in HCC cells was determined by immunofluorescence^30^. HCC cells were grown on glass cover slips to 20–30% confluency and then fixed, permeabilized, blocked, and incubated with TGM2 antibody overnight at 4 °C. The slides were subsequently washed and incubated with Alexa fluor 555-conjugated secondary antibody (Beyotime Biotechnology). To visualise nuclear details, the cells were counterstained using 4′-6-diamidino-2-phenylindole (DAPI), and fluorescence microscopic examination (Olympus) was performed.

### Immunohistochemistry

Tumour specimens were removed, placed in 4% paraformaldehyde, and sectioned (5 μm). The slides were incubated with primary antibodies overnight, and a two-step visualisation system was applied (EnVision; Agilent, Santa Clara, CA, USA). After counterstaining (Mayer’s haematoxylin) and coverslipping, photos were taken under a light microscope (200×), selecting five fields at random for uniform capture^28^.

### Quantitative real-time polymerase chain reaction (qRT-PCR)

RNA was isolated from HCC cells after co-culture experimentation, using TRIzol Reagent (Invitrogen, Waltham, MA, USA) as directed by the manufacturer. Complementary DNA was then synthesised (Reserve Transcription System; Promega Corp, Madison, WI, USA), acting as a template for RT-PCR, employing gene-specific primers and SYBR Premix Ex Taq reagent (Takara Bio Inc, Kyoto, Japan). Relative mRNA expression levels by genes of interest were determined via qRT-PCR (7500 Real-Time PCR System; Applied Biosystems). Primers for genes of HIF-1a and the 18S housekeeping gene were commercially procured (Sangon Biotech Co., Ltd., Shanghai, China). Relative mRNA levels were calculated using the –ΔΔCt method (18S housekeeping gene serving as control) and were expressed as 2(−ΔΔCt).

RT-PCR primers are shown in Table 1.

### Enzyme-linked immunosorbent assay (ELISA)

Concentrations of IL-6 and TGF-β1 in cell supernatants were quantified using ELISA kits (USCN Life Science Inc., Wuhan, China), performing assays as directed by the manufacturer.

### Detection of dissolved oxygen concentration

Concentrations of dissolved oxygen in cell supernatants were quantified using portable dissolved oxygen metres (JPB-607A; Rex, Shanghai, China). Assays were performed according to the manufacturer’s instructions.

### The Cancer Genome Atlas (TCGA) data analysis

For mRNA expression analysis, the RNA-seq V2 data was obtained from TCGA database^32^. The normalised RNA-Seq by expectation maximisation value of TGM2 expression in 46 HCC tissues and adjacent normal tissues were extracted and merged, and paired student’s *t*-test was preformed to verify the statistical significance. The Pearson’s correlation was calculated to test the link between TGM2 mRNA expression and the EMT marker E-cadherin and Vimentin expression in 371 HCC tissues.

### Statistical analysis

Cumulative recurrence-free and overall survival rates were estimated by Kaplan–Meier method, evaluating between-group differences via log-rank test. In vitro results were expressed as mean ± SE. All computations relied on standard software (SPSS v19.0 for Windows; IBM, Armonk, NY, USA), setting significance at *P* < 0.05.

## Electronic supplementary material

Supplementary file

## Data Availability

Data set corresponding to quantitative proteomics have been deposited to the ProteomeXchange Consortium via the PRIDE^33^ partner repository with the data set identifier PXD01103. Additional data that support the findings of this study are available in supplementary information and from the corresponding author upon request.
