# Supplementary material for: Activated hepatic stellate cells promote epithelial-to-mesenchymal transition in hepatocellular carcinoma through transglutaminase 2-induced pseudohypoxia
Source: Commun Biol. 2018 Oct 25;1:168. doi: 10.1038/s42003-018-0177-5 (PMC6202353; doi:10.1038/s42003-018-0177-5)
Supplement: Supplementary file 1 — Supplementary file [file 42003_2018_177_MOESM1_ESM.pdf]

Supplementary information

Supplementary Figure 1

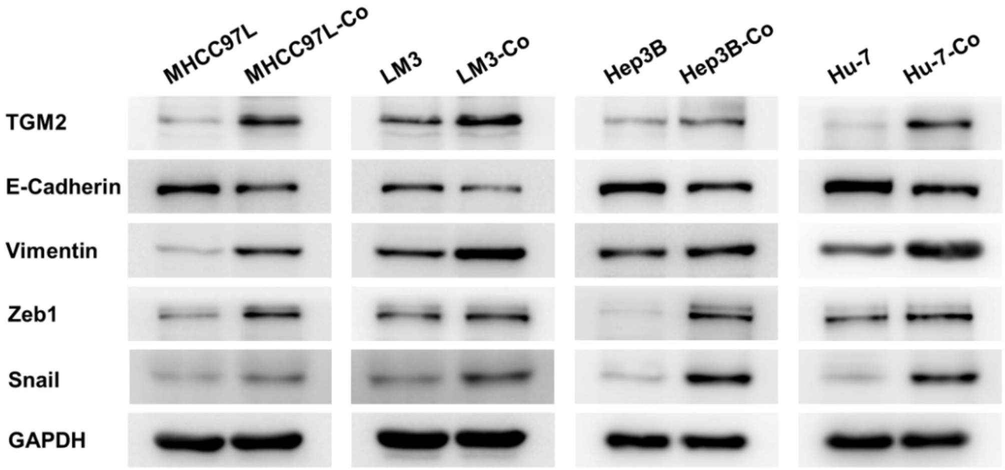

Supplementary Figure 1. Activated hepatic stellate cells promote EMT and TGM2 upregulation in HCC cells. Lower E-cadherin and higher TGM2, Vimentin, Zeb1 and Snail expression levels in HCC cells (MHCC97L, LM3, Hep3B and Hu-7) co-cultured with hepatic stellate cells (western blotting).

Supplementary Figure 2

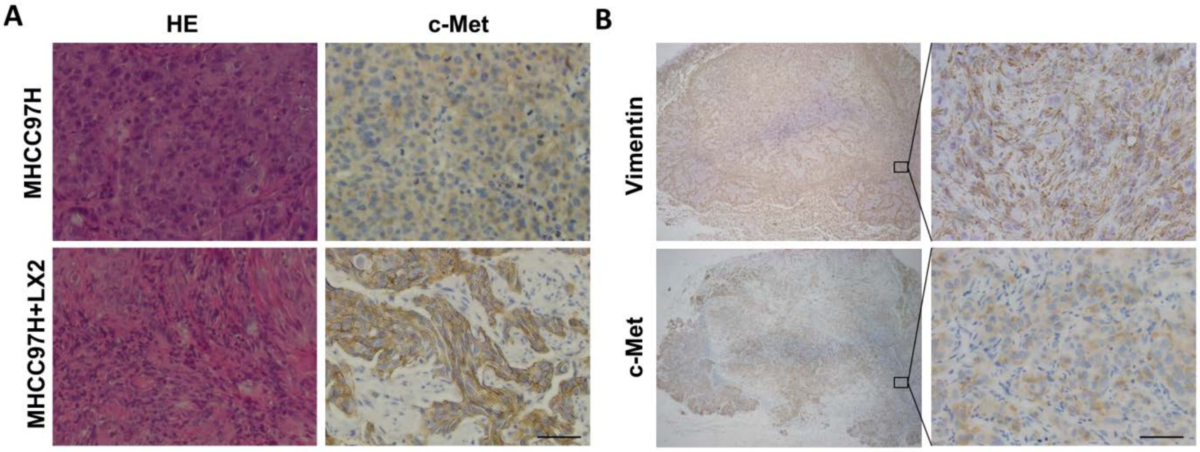

Supplementary Figure 2. c-Met expression in tumours spawned by combined injection of HCC cells and hepatic stellate cells into nude mice. (A) Haematoxylin and eosin (HE)-stained images and immunostaining of c-Met are shown (scale bar, 50μm). (B) Immunostaining images of Vimentin and c-Met are shown in the serial sections (scale bar, 50μm).

Supplementary Figure 3

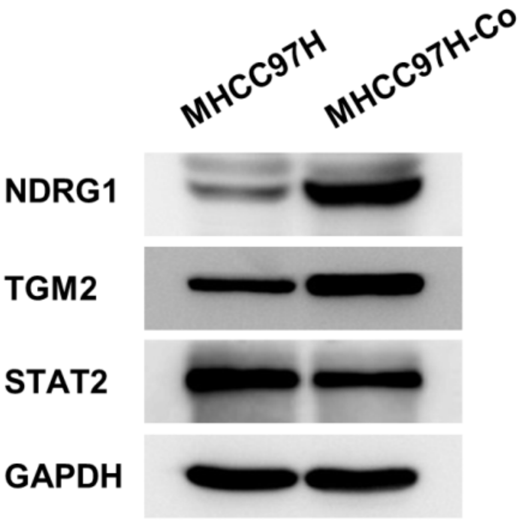

Supplementary Figure 3. Accuracy of mass spectra results was validated of NDRG1, TGM2, and STAT2 (western blotting).

Supplementary Figure 4

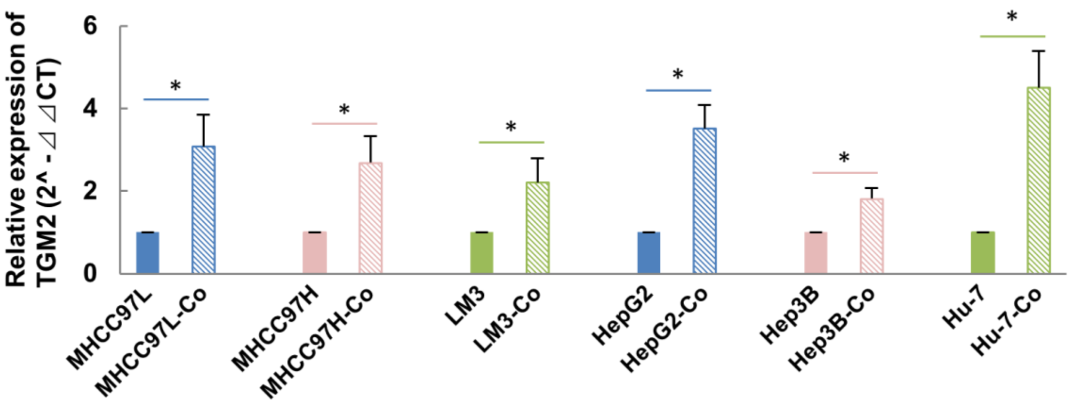

Supplementary Figure 4. Activated hepatic stellate cells promote TGM2 mRNA upregulation in HCC cells. Higher TGM2 expression at mRNA levels in HCC cells (MHCC97L, MHCC97H, LM3, HepG2, Hep3B and Hu-7) co-cultured with hepatic stellate cells (quantitative real-time Polymerase Chain Reaction).

Supplementary Figure 5

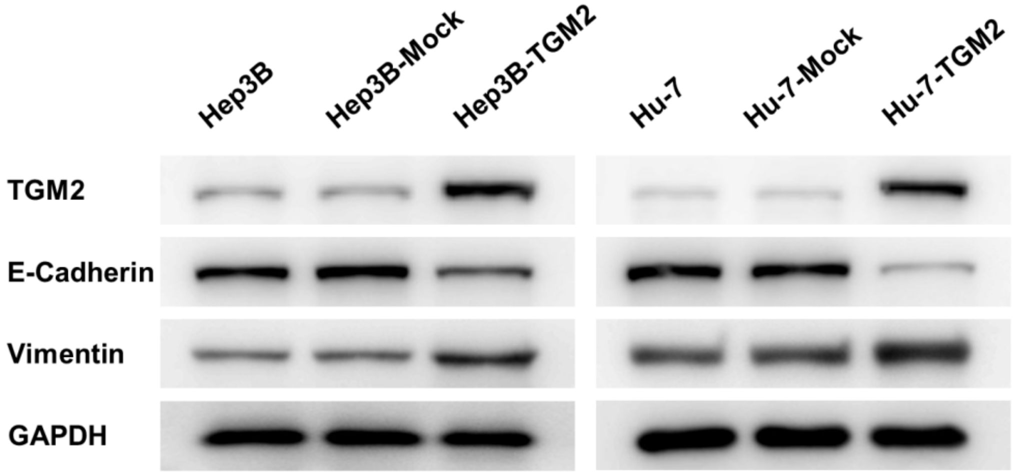

Supplementary Figure 5. TGM2 upregulation promotes EMT in HCC cells. Lower E-cadherin and higher Vimentin expression levels in Hep3B-TGM2 OE and Hu-7-TGM2 OE cells, compared with Hep3B-Mock and Hu-7-Mock cells, respectively (western blotting).

Supplementary Figure 6

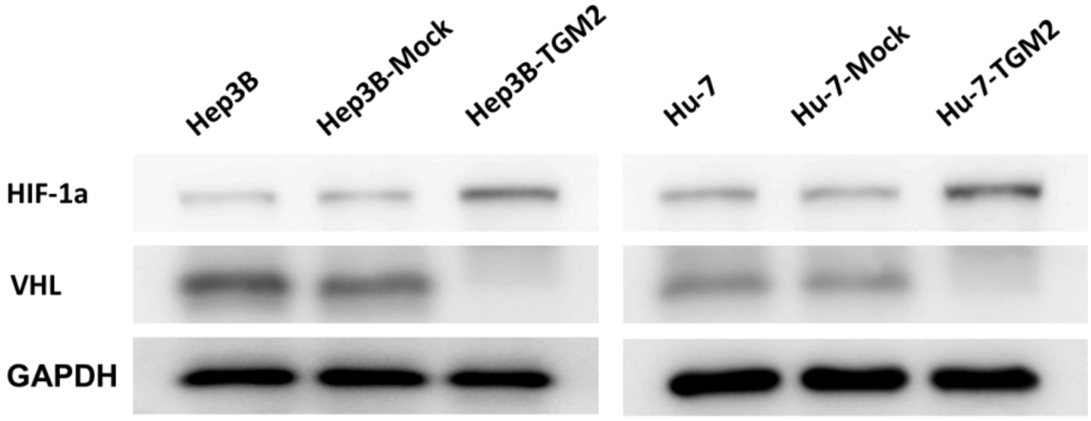

Supplementary Figure 6. TGM2 upregulation results in the accumulation of HIF-1a in HCC cells Higher HIF-1a and lower VHL expression levels in Hep3B-TGM2 OE and Hu-7-TGM2 OE cells, compared with Hep3B-Mock and Hu-7-Mock cells, respectively (western blotting).

Supplementary Figure 7

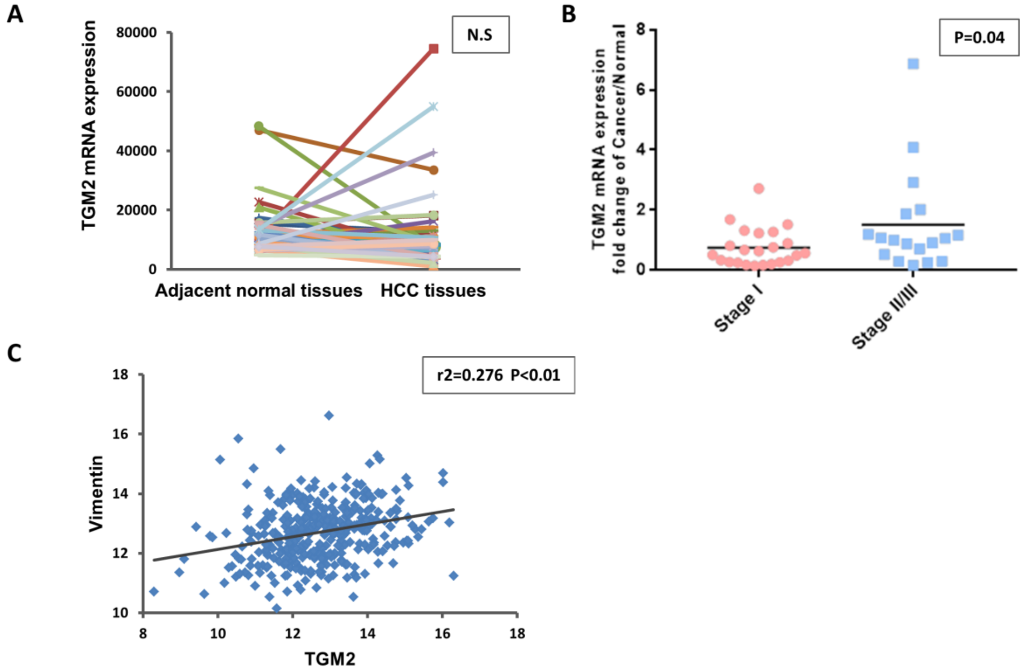

Supplementary Figure 7. (A) The expression of TGM2 in HCC tissues and adjacent normal tissues from TCGA (no significance). (B) The TGM2 expression radio of HCC to adjacent normal tissues in HCC with different TNM stages from TCGA (P=0.04). (C) The Spearman's correlation analysis between Vimentin and TGM2 expression in 371 HCC samples from TCGA (P<0.01).

Supplementary Figure 8

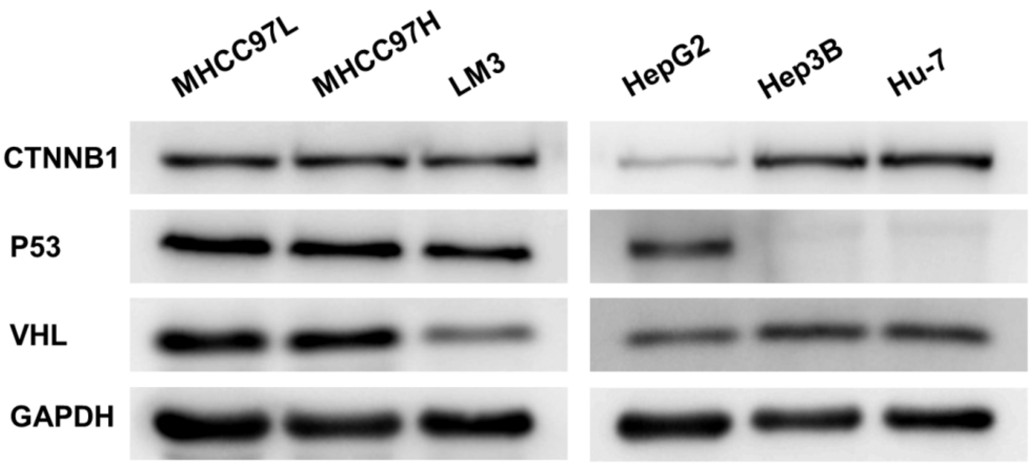

Supplementary Figure 8. CTNNB1, P53 and VHL expression in HCC cell lines (MHCC97L, MHCC97H, LM3, HepG2, Hep3B and Hu-7) (western blotting).

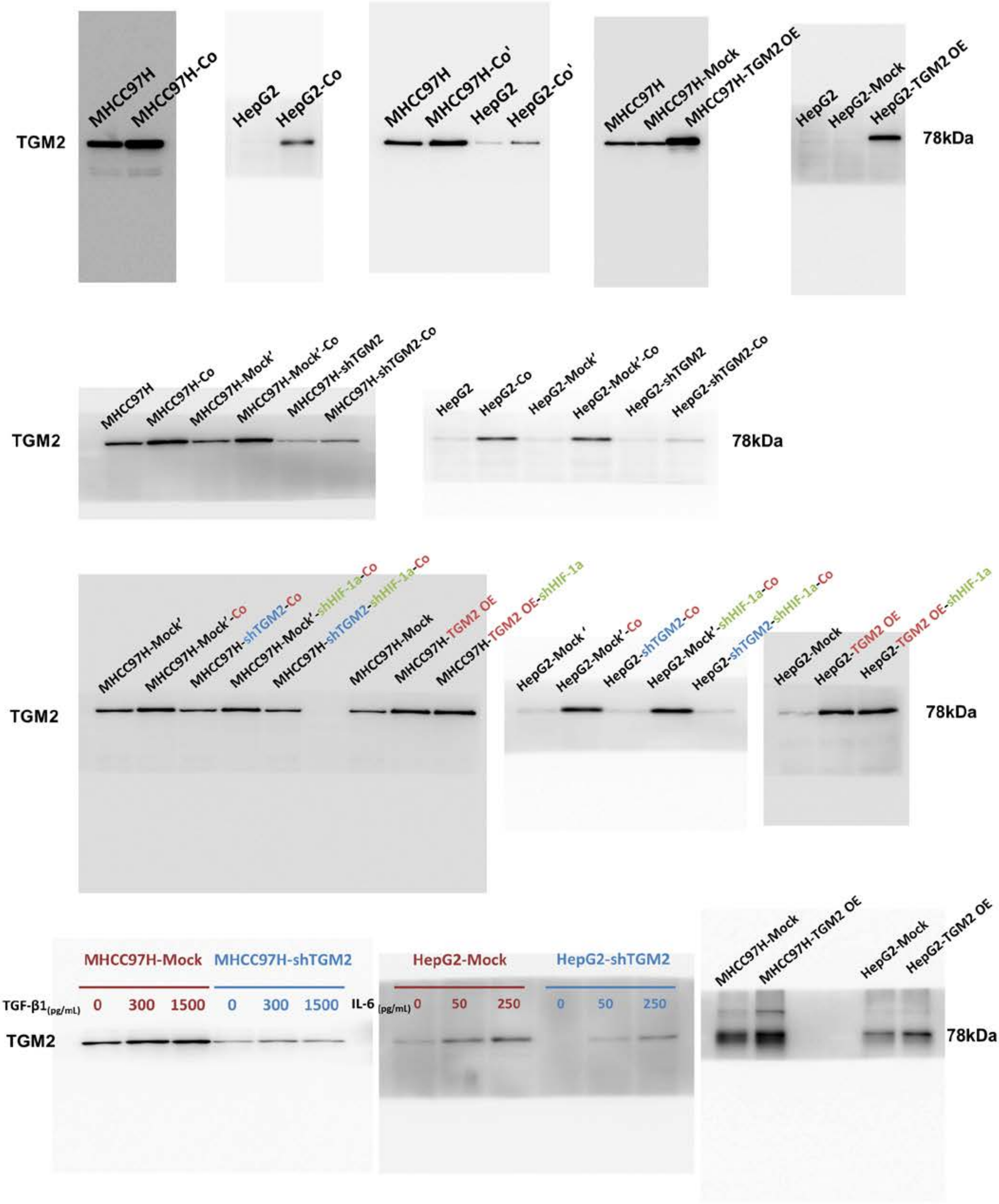

Supplementary Figure 9. Original blot images corresponding to western blots presented in the main figures.

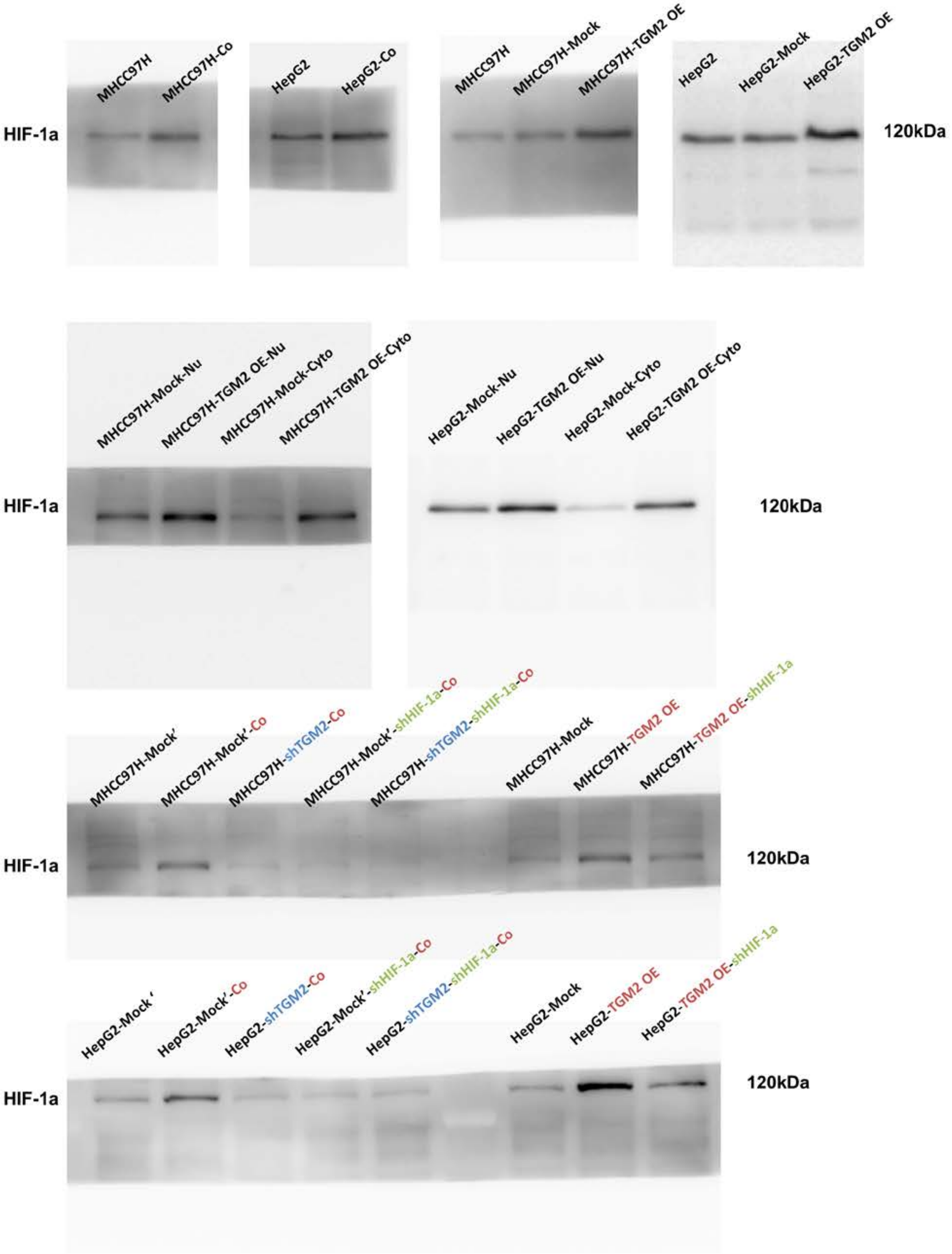

Supplementary Figure 9 continued

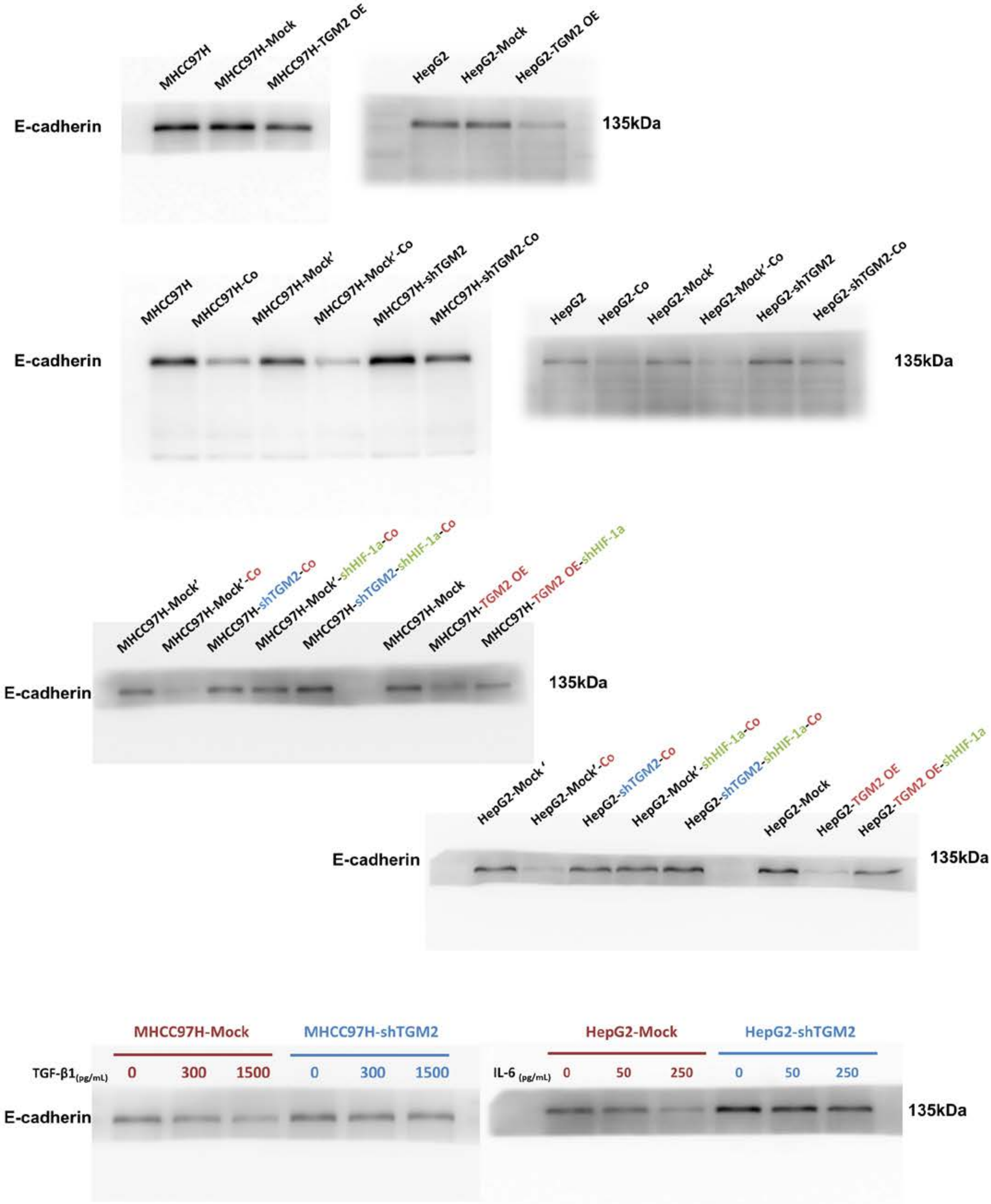

Supplementary Figure 9 continued

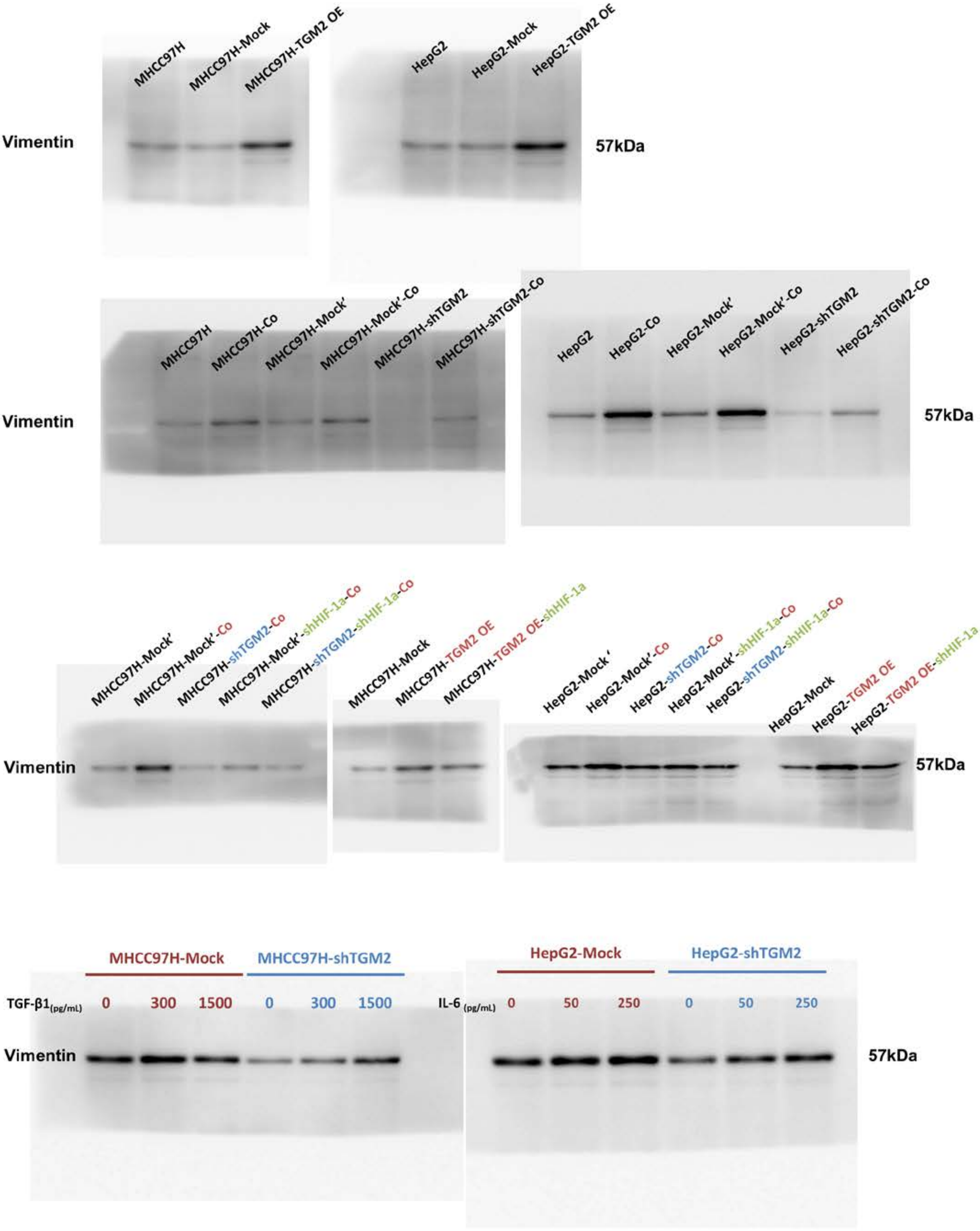

Supplementary Figure 9 continued

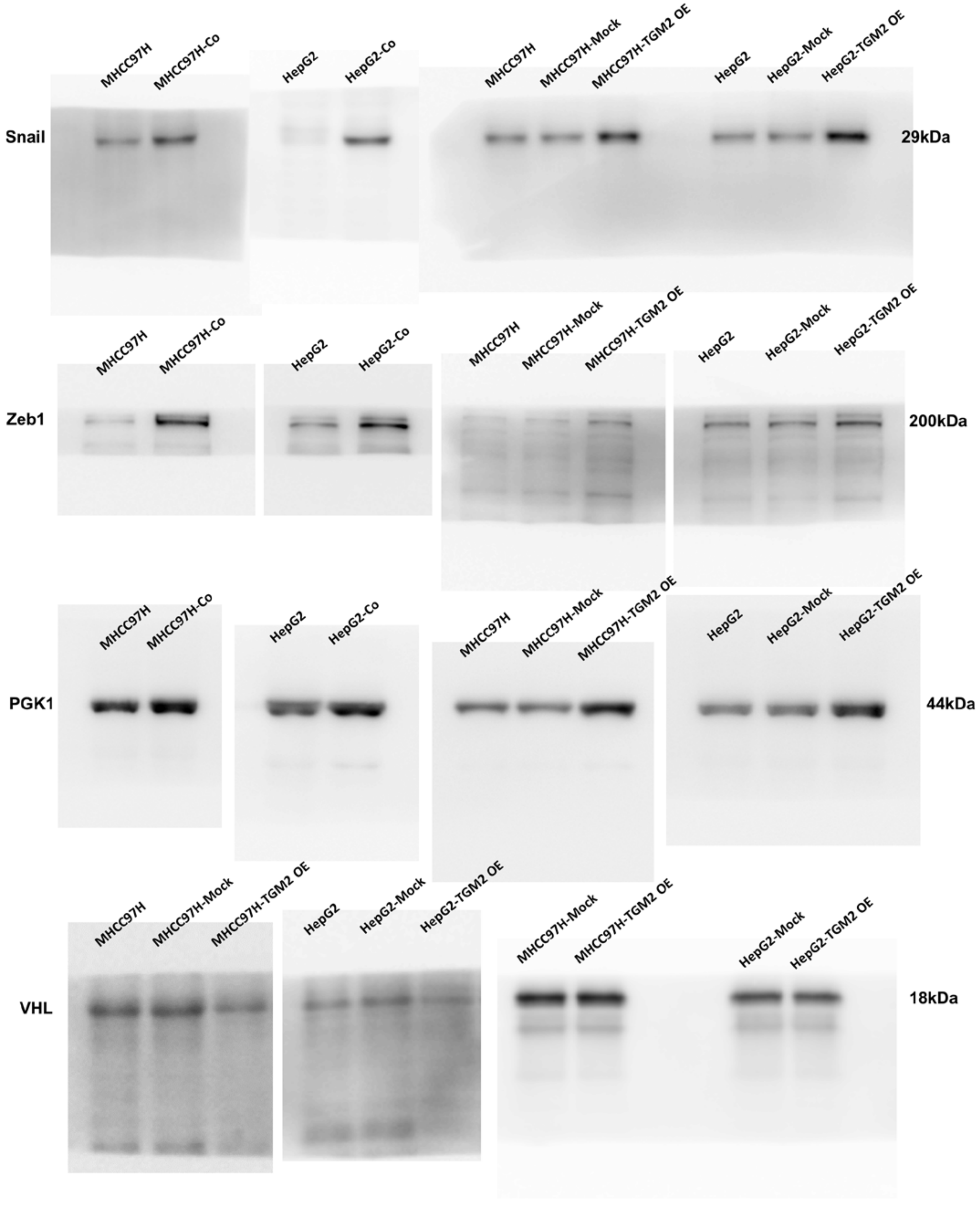

Supplementary Figure 9 continued

Supplementary Table 1. List of the up-regulated Proteins

Accession	Symbol	Ratio	Accession	Symbol	Ratio
Q9Y2L5	TPPC8	5.57	Q9BVM2	DPCD	2.20
O95208	EPN2	5.26	Q6PI78	TMM65	2.20
Q9Y3B7	RM11	4.98	Q02952	AKA12	2.19
Q8TD19	NEK9	4.75	P08648	ITA5	2.17
Q92597	NDRG1	4.63	Q86X76	NIT1	2.16
Q16790	CAH9	4.49	Q9Y6N5	SQRD	2.16
O00584	RNT2	4.25	P05362	ICAM1	2.12
P12694	ODBA	3.70	Q5SSJ5	HP1B3	2.10
P00450	CERU	3.36	Q16795	NDUA9	2.10
P19793	RXRA	3.24	P12109	CO6A1	2.06
P15941	MUC1	3.15	P10451	SPP1	2.03
O43678	NDUA2	3.05	Q96JD6	AKCL2	2.02
P09972	ALDOC	2.91	O15427	MOT4	2.01
Q6GMV2	SMYD5	2.88	Q5MNZ6	WIPI3	2.01
P50895	BCAM	2.86	Q16134	ETFD	1.99
Q02318	CP27A	2.68	O95747	OXSR1	1.96
P04179	SODM	2.65	Q15047	SETB1	1.94
O00469	PLOD2	2.59	Q07954	LRP1	1.93
P21980	TGM2	2.58	Q92797	SYMPK	1.92
O15460	P4HA2	2.50	O60936	NOL3	1.88
P55263	ADK	2.50	Q13492	PICAL	1.86
P13674	P4HA1	2.49	Q68CZ2	TENS3	1.85
Q96HE7	ERO1A	2.45	Q9Y376	CAB39	1.85
P09104	ENOG	2.45	Q96J84	KIRR1	1.84
Q9P2R7	SUCB1	2.41	P50224	ST1A3	1.84
P51580	TPMT	2.33	P04080	CYTB	1.80
C9JLW8	F195B	2.32	P11166	GTR1	1.79
Q96A26	F162A	2.31	P00558	PGK1	1.77
Q03426	KIME	2.29	O43809	CPSF5	1.77
Q9BQE5	APOL2	2.23	Q5TBC7	B2L15	1.77

Supplementary Table 1 (continue)

Accession	Symbol	Ratio	Accession	Symbol	Ratio
P34949	MPI	1.76	O75886	STAM2	1.60
P09493	TPM1	1.76	P63220	RS21	1.60
Q14195	DPYL3	1.76	P00403	COX2	1.59
O43760	SNG2	1.75	Q9BVK6	TMED9	1.57
Q6UXV4	APOOL	1.73	Q9Y3E5	PTH2	1.55
P35914	HMGCL	1.71	P02751	FINC	1.55
Q9Y487	VPP2	1.71	P46736	BRCC3	1.55
Q08722	CD47	1.71	P10619	PPGB	1.55
P07093	GDN	1.71	P27105	STOM	1.54
Q12907	LMAN2	1.70	Q14764	MVP	1.54
P29966	MARCS	1.70	Q6ZMU5	TRI72	1.53
P06737	PYGL	1.69	P53004	BIEA	1.53
Q12986	NFX1	1.69	Q04446	GLGB	1.52
P13807	GYS1	1.68	O95834	EMAL2	1.52
P12235	ADT1	1.66	Q86W42	THOC6	1.52
P06703	S10A6	1.65	P00338	LDHA	1.52
P21912	DHSB	1.64	Q8IVF2	AHNK2	1.52
Q96C23	GALM	1.63	Q8IWE2	NXP20	1.51
Q16666	IF16	1.62	P06396	GELS	1.51
P49773	HINT1	1.61	Q13287	NMI	1.50
P56545	CTBP2	1.61	Q96Q06	PLIN4	1.50
P04424	ARLY	1.60	P46977	STT3A	1.50
P29590	PML	1.60	Q9BQB6	VKOR1	1.50

Supplementary Table 2. List of the down-regulated Proteins

Accession	Symbol	Ratio	Accession	Symbol	Ratio
O14735	CDIPT	9.62	Q9NR56	MBNL1	1.78
Q13190	STX5	7.23	P20290	BTF3	1.77
P35527	K1C9	3.32	P02792	FRIL	1.74
P08579	RU2B	3.23	Q9NWY4	CD027	1.74
P52630	STAT2	3.19	Q9UI12	VATH	1.73
P55081	MFAP1	3.04	Q13206	DDX10	1.73
Q13823	NOG2	3.02	P13073	COX41	1.68
Q9BYN0	SRXN1	2.99	P98172	EFNB1	1.68
Q08170	SRSF4	2.84	P10109	ADX	1.67
P18615	NELFE	2.68	Q96D71	REPS1	1.66
P42345	MTOR	2.52	O43818	U3IP2	1.66
Q9Y383	LC7L2	2.45	P51532	SMCA4	1.64
Q6PD62	CTR9	2.38	Q8N6R0	MET13	1.64
P04264	K2C1	2.25	Q8NB90	SPAT5	1.63
Q13501	SQSTM	2.24	P62495	ERF1	1.62
Q9GZN1	ARP6	2.23	Q92887	MRP2	1.62
P52292	IMA2	2.21	P29144	TPP2	1.61
Q9BZX2	UCK2	2.20	O00488	ZN593	1.60
Q9UBU9	NXF1	2.20	Q8IUE6	H2A2B	1.58
O95183	VAMP5	2.10	Q9NXR1	NDE1	1.58
P15559	NQO1	2.04	O43657	TSN6	1.57
Q712K3	UB2R2	2.00	Q9Y4A5	TRRAP	1.57
Q96T88	UHRF1	1.98	Q9Y5Q9	TF3C3	1.56
Q8IXT5	RB12B	1.97	P52732	KIF11	1.55
Q96EI5	TCAL4	1.93	O00762	UBE2C	1.55
Q8IUH3	RBM45	1.87	P35249	RFC4	1.55
P33316	DUT	1.85	P40938	RFC3	1.54
Q8N335	GPD1L	1.84	P23258	TBG1	1.54
Q9H2U1	DHX36	1.84	Q6VEQ5	WASH2	1.53
O95551	TYDP2	1.80	Q969S3	ZN622	1.53

Supplementary Table 2 (continue)

Accession	Symbol	Ratio	Accession	Symbol	Ratio
Q10713	MPPA	1.53	P48681	NEST	1.52
Q96KB5	TOPK	1.52	Q5M775	CYTSB	1.51
O94804	STK10	1.52	Q9BQC3	DPH2	1.50
Q15758	AAAT	1.52	Q9H0B6	KLC2	1.50
Q5T3I0	GPTC4	1.52	Q6IQ49	SDE2	1.50

Supplementary Table 3. Primary antibodies for western blot, immunoprecipitation, immunohistochemistry and immunofluorescence

Antibody	WB	IHC	IF	IP	Specificity	Company
TGM2	1:2000	1:500	1:500	1:50	monoclonal	Abcam
E-cadherin	1:1000	1:200	-	-	monoclonal	CST
Vimentin	1:1000	1:200	-	-	monoclonal	CST
HIF-1a	1:1000	1:200	-	-	monoclonal	Abcam
PGK1	1:1000	-	-	-	monoclonal	Abcam
ENOG	1:1000	-	-	-	monoclonal	Abcam
LDHA	1:1000	-	-	-	monoclonal	CST
Hydroxy-HIF-1a	1:1000	-	-	-	monoclonal	CST
VHL	1:1000	-	-	-	monoclonal	Abcam
NDRG1	1:1000	-	-	-	polyclonal	Aviva Systems Biology
STAT2	1:1000	-	-	-	monoclonal	CST
GAPDH	1:1000	-	-	-	monoclonal	CST
Tubulin	1:1000	-	-	-	monoclonal	CST
Histone H3	1:2000	-	-	-	monoclonal	CST

Supplementary Methods

Protein extraction, isotopic dimethylation labeling and LC/MS-MS analysis

The whole-cell lysates of the MHCC97H cells with or without co-cultured with LX2 cells were extracted using lysis buffer containing 7M urea (Sigma), 2M thiourea (Sigma) and protease inhibitor (Roche) by continuous votex at 4°C for 1 hr. After centrifugation at 15000 g for 30 min at 4°C, the protein abundance supernatant was determined using Bradford method, then aliquoted and stored at -80°C. Next, 100μg of protein from each sample was transferred into 10K ultrafiltration tube (Millipore). These proteins were reduced with 10 mM dithiothreitol (60 min, 56°C) and alkylated with 12 mM iodoacetamide in darkness (45 min, 37°C). To remove contaminants, samples were centrifuged by adding 100mM TEAB for three times. 2 μg trypsin (Promega, Madison, WI, USA) was added for overnight digestion at 37°C. After that, tryptic peptides from two samples were isotopically dimethylated as previous study [1]. Briefly, 4μl of 4% CH_2O (Light) or 4% CD_2O (Heavy) was added followed by 4μl of 600 mM NaBH3CN. The mixture was incubated for 1 h at room temperature. The reaction was quenched with ammonia. The differentially labeled samples were then pooled and desalted using Sep-Pak Vac C18 (Waters, Milford, MA, USA).

The peptide mixture was lyophilized again and dissolved in 20mM ammonium formate in water, pH10.0, adjusted with ammonium hydroxide, and then fractionated by high pH separation using Ultimate 3000 system (ThermoFisher scientific, MA, USA) connected to a reverse phase column (XBridge C18 column, 4.6mm x 250 mm, 5μm, (Waters Corporation, MA, USA). High pH separation was performed using a linear gradient. Starting from 5% B to 45% B in 40 min (B: 20mM ammonium formate in 80% ACN, pH 10.0, adjusted with ammonium hydroxide). 15 offline fractions were collected and dried in a vacuum concentrator. Each fractionation was redissolved in 0.1% formic acid and 5% acetonitrile and injected into the NanoLC-1D Plus HPLC (ABSCIEX, Eksigent Technologies, Dublin, CA) coupled online with a TripleTOF 5600 (AB SCIEX, Framingham, USA). The peptide mixture was separated on a ZORBAX 300SB-C18 column (5 μm particle size, 0.1 mm × 150 mm length, and 300 Å pore size, Agilent, Palo Alto, USA) with a 85 min linear gradient of 5% to 36.5% acetonitrile in 0.1% formic acid. The flow rate was 0.3 μL/min. The mass range was set as 400−1800 m/z. The top 15 precursor peaks were selected for CID.

Three independent experiments were repeated.

Bioinformatics Analysis of Differentially Expressed Proteins

Protein identification and quantification for experiments was carried out using ProteinPilot 4.2 software (Applied Biosystems; MDS-Sciex). Database search was performed against a target-decoy database constructed based on SwissProt Human. Only proteins identified with at least 95% confidence were reported.

To identify proteins that were either up- or down-regulated, the ratios of proteins identified in three independent analyses were averaged. The threshold value that was set for significant regulated proteins in the present study was 1.5 fold.

The bioinformatics analysis of the differentially expressed proteins was performed with Ingenuity Pathways Analysis (IPA) software (2016 summer, Ingenuity Systems, Redwood City, CA, http://www.ingenuity.com). The differentially expressed proteins and the corresponding expression values were uploaded into the software and assigned into different molecular and cellular functional classes based upon the underlying biological evidence from the curated Ingenuity Pathways Knowledge Base. Functional networks of these proteins were algorithmically generated based on their connectivity and ranked according to relevancy to the proteins in the input dataset. Canonical pathway analysis identified the pathways, from the IPA library of canonical pathways, which were most significant to the input dataset. The main biological functions of the differentially expressed proteins were also collected from Uniprot protein knowledge database (http://www.uniprot.org) and PubMed (http://www.ncbi.nlm.nih.gov).

Reference

1. Boersema PJ, Aye TT, Van Veen TA, et al. Triplex protein quantification based on stable isotope labeling by peptide dimethylation applied to cell and tissue lysates. Proteomics. 2008; 8(22): 4624-4632.
